# Changes in childhood experimentation with, and exposure to, tobacco and e-cigarettes and perceived smoking norms: a repeated cross-sectional study of 10–11 year olds’ in Wales

**DOI:** 10.1186/s12889-021-12004-z

**Published:** 2021-10-23

**Authors:** Britt Hallingberg, Lianna Angel, Rachel Brown, Lauren Copeland, Linsay Gray, Jordan Van Godwin, Graham Moore

**Affiliations:** 1 DECIPHer, School of Social Sciences, Cardiff University, Wales, UK; 2 Cardiff School of Sport & Health Sciences, Cardiff Metropolitan University, Wales, UK; 3 MRC/CSO Social and Public Health Sciences Unit, University of Glasgow, Scotland, UK

**Keywords:** E-cigarettes, Tobacco, Smoking, Children, Parents, Exposure, Smoking norms, Perceptions

## Abstract

**Background:**

Today’s primary school children have grown up in a climate of strong smoking restrictions, decreasing tobacco use, and the emergence of e-cigarettes. Children’s exposure to tobacco declined substantially in years following the introduction of smoke-free legislation, with smoking uptake and perceived smoking norms declining. There is debate regarding whether emergence of e-cigarettes may interrupt trends in children’s smoking perceptions, or offer a means for adults to limit children’s exposure to tobacco. This study examines change in children’s tobacco and e-cigarettes experimentation (ever use), exposure to secondhand smoking and vaping, and perceived smoking norms.

**Methods:**

Data from four, repeat cross-sectional surveys of Year 6 primary school pupils (age 10–11 years) in Wales in 2007, 2008, 2014 and 2019 (*n* = 6741) were combined. E-cigarette use and perceptions were included in 2014 and 2019 surveys. Analyses used binary logistic regression analyses, adjusted for school-level clustering.

**Results:**

Child tobacco experimentation and most indicators of exposure to tobacco smoke indicated a graded decreasing trend over time from 2007 to 2019. Exposure to e-cigarettes increased from 2014 to 2019, as did pupil awareness of e-cigarettes (OR = 2.56, 95%CI = 2.12–3.10), and parental use (OR = 1.26, 95%CI = 1.00–1.57). A decrease in child e-cigarette experimentation was not significant (OR = 0.80, 95%CI = 0.57–1.13). Children’s normative perceptions for smoking by adults and children indicated a graded decrease over time (OR = 0.66, 95%CI = 0.54–0.80; OR = 0.69, 95%CI = 0.55–0.86; respectively from 2014 to 2019). However, fewer reported disapproval of people smoking around them in 2019 relative to 2014 (OR = 0.68, 95%CI = 0.53–0.88). Higher exposure to tobacco cigarettes and e-cigarettes in public places, cars and households were associated with favourable normative perceptions for tobacco smoking; however in models adjusted for exposure to both associations of e-cigarette exposure were attenuated.

**Conclusion:**

Children’s experimentation with and exposure to tobacco, and their perceptions of smoking as a normative behaviour, have continued to decline alongside growth in exposure to e-cigarettes. Although a large majority of pupils reported they minded people smoking around them, there was some evidence of diminishing disapproval of secondhand smoke since 2007. Further research is needed to understand whether use of e-cigarettes in cars and homes is displacing prior smoking or being introduced into environments where smoking had been eliminated.

**Supplementary Information:**

The online version contains supplementary material available at 10.1186/s12889-021-12004-z.

## Background

Over the past 20 years, legislation in many high-income countries has increasingly restricted where and when smoking can take place (1). In the United Kingdom, legislation which prohibited tobacco (cigarette) smoking in enclosed public places and workplaces was recently voted by the Royal Society for Public Health as the biggest public health achievement of the twenty-first century (2). This was implemented with the primary aim of reducing exposure to smoke among groups including those working in the hospitality industry (3), and evidence indicated that it was successful in achieving these aims. However, much attention also focused on its impacts on childhood exposure to tobacco. In particular, in opposing the legislation, some advocated the ‘displacement’ hypothesis, promoting concerns that public smoking bans would have the perverse effect of displacing smoking into the home, thus increasing children’s exposure to secondhand smoke (4).

A large body of international evidence finds that this did not occur (5). For example, a meta-analysis of the impact of public smoking bans on children’s exposure to secondhand smoke at home revealed an overall decrease in exposure (1). Rather than displacing smoking into the home, legislation perhaps contributed further to denormalization of smoking in the presence of children, leading to reduced secondhand smoke exposure (6–9), and changing perceptions of smoking as a ‘normal’ behaviour (10). Throughout the past two decades of increasing regulation of smoking in the UK, youth smoking uptake has declined, while anti-smoking attitudes and normative perceptions among youth have hardened (11). In the years following legislation on smoking public places, surveys in the UK and beyond showed strong public support for further action to limit children’s exposure to tobacco (12–14), with smoking in cars carrying children banned across the UK from 2015 (15), and restrictions of smoking in school grounds and playground from 2021 (16, 17).

During the period since the introduction of smoke-free legislation, e-cigarettes have emerged and gained traction within UK markets, with rapid growth in use primarily by adult smokers and ex-smokers (18) from around 2011, followed by a plateauing since 2013 (19, 20). Although e-cigarettes are not harmless (19), there is general consensus that they are less harmful than tobacco cigarettes (21) and may be beneficial for quitting smoking. In a UK randomised trial, e-cigarettes combined with behavioural support were almost twice as effective as a cessation tool compared to nicotine replacement therapy (NRT) with behavioural support (22). A recent randomised control trial found that female smokers improved their vascular health within 1 month of switching from tobacco to e-cigarettes, irrespective of nicotine content (23). Hence, e-cigarettes may offer important harm reduction potential when used by smokers as a means of quitting smoking.

However, concerns regarding the emergence of e-cigarettes have centred largely on debates regarding effects on young people, in particular via their acting as a new gateway to nicotine addiction (19, 24), or renormalising smoking (25–27). Substantial debate has centred on whether e-cigarettes should be regulated in the same ways as tobacco, including whether their use in public places should be prohibited. In 2015 for example, the Welsh Government attempted unsuccessfully to extend legislation banning use of tobacco cigarettes in public spaces to include e-cigarettes, citing concerns that e-cigarettes will renormalise smoking (28). In the US, 22 states/territories and over 900 municipalities, include e-cigarettes within legislation prohibiting their use in public places and workplaces where smoking is banned (29, 30). Recent findings from a cross-sectional nationally representative school survey in the US found increased exposure to e-cigarette aerosol in public places was associated with increased susceptibility to overestimating peer tobacco use (25).

A growing body of longitudinal studies finds that young people’s use of e-cigarettes is associated with subsequent smoking (31). However, causality remains contested, with residual confounding remaining a likely partial explanation for these trends (32). Youth smoking rates and attitudes in favour of smoking continued to decline during the emergence of e-cigarettes (11), while our analyses of survey data and qualitative data on primary school pupils in Wales from the present study indicate that parental vaping is associated with perceived smoking norms only where it occurs alongside smoking (33, 34). Indeed, children whose parents used e-cigarettes were more likely to perceive these as devices adults used to stop smoking, with perceiving e-cigarettes as a means of giving up smoking associated with lower reported susceptibility to smoking (33, 34).

While use of e-cigarettes in public places has received significant policy attention, e-cigarette use in private spaces such as homes and cars has received less attention. Our previous research indicated that while smokers with children had increasingly restricted smoking in their home, almost half of children with a parent who smoked continued to report that smoking was allowed in their home in 2014 (15). Although it remains contested whether secondhand vaping poses health risks to children, few health risks have been identified to date (35). In Scotland, parents of infants in disadvantaged areas emphasised the value of using e-cigarettes in the home when they lacked direct access to outside space or could not leave their homes to smoke (36). Secondary school pupils in England reported they preferred adult’s use of e-cigarette use in the home over tobacco smoking (37), although parents employed strategies to protect young people from e-cigarette vapour by restricting use both indoors and outdoors. Among parents who remain addicted to nicotine, but face barriers to maintaining smoke-free home environments, e-cigarettes may act as a means of limiting childhood exposure to more harmful tobacco smoke (38, 39).

There remains scant research on primary school pupils’ perceptions of, and exposure to e-cigarettes (7, 30), as well as how this relates to perceived smoking norms. As discussed above, debates surrounding the increased visibility of e-cigarettes have focused on the potential for them to renormalise smoking through the appearance of a smoking-like behaviour being accepted among others (26). However, as these pupils are the first generation to be born in the years immediately following smoke-free legislation (when smoking in the population has been decreasing) alongside the parallel emergence of e-cigarettes, understanding experiences and perceptions of tobacco among this cohort of children is important in shaping contemporary policy. The aims of the current study are to examine changes over time (from 2007 to 2019 for variables relating to tobacco and from 2014 to 2019 for those related to e-cigarettes) in:

1. children’s experimentation with tobacco and e-cigarette use, and future use intentions;
2. children’s exposure to tobacco smoke and vaping in public places and private spaces;
3. children’s perceived smoking norms.

Finally, we examine the relationships of children’s exposure to secondhand tobacco smoke and e-cigarette vapour with perceived smoking norms.

## Methods

### Design and sampling

Data were analysed from primary school surveys conducted in 2007, 2008, 2014 and 2019, which were designed to be nationally representative of Year 6 pupils (i.e. 10–11 year olds) in schools in Wales and collected in a classroom setting by trained researchers (8, 40–42). Schools were originally selected through stratified random sampling to ensure representation of all local authorities in Wales, and high/low deprivation (indicated by Free School Meal Entitlement, a proxy for socioeconomic status) (40). The 2007 and 2008 surveys included the same schools, who were recruited for two surveys as part of a before and after study of children’s exposure to secondhand smoke (CHETS Wales (8)), modelled on a similar study in Scotland (6). For the 2014 survey, the same schools were approached, with those who chose not to take part again replaced by a school randomly selected from the same strata. This was repeated in 2019. More detail on study sampling and data collection protocols are described in our earlier open access publications (34, 40, 41).

In all surveys, a signed agreement was obtained from the Head Teacher of participating schools. Parents were given the chance to opt their child out by returning a freepost opt-out slip. Pupils were given the option to take part on the day. Study protocols for each survey were approved by the Cardiff University School of Social Sciences Research Ethics Committee. In 2018, for the mixed methods study of which the survey formed a part, pupils in one school provided input into the development of study materials. Several young people in the target age group known to the research team assessed draft questionnaires for readability and timing and provided feedback on questions. While many items were fixed due to the focus on changes over time, this enabled redundant items to be removed and any newer items to be tested, in order to maximise completeness of data once the survey was undertaken.

### Measures

#### Demographics

Gender was measured by asking “are you a i) boy, ii) girl, [plus iii) prefer to self-describe, iv) prefer not to say” in the 2019 survey only]. Pupil affluence was measured using the Family Affluence Scale (FAS) (43). The 2007–2014 surveys used a shorter FAS scale comprising items on computer and car ownership, bedroom occupancy and family holidays (44). Items on dishwasher and bathrooms in the home were later added. In 2019, the item regarding ‘family holidays’ was not used due to confusion regarding what constituted a holiday during Public Involvement work with young people.

#### Pupil awareness of e-cigarettes, ever smoking/vaping and perceived susceptibility to future smoking and vaping

Items relating to e-cigarettes were asked in 2014 and 2019 only. E-cigarettes were defined as ‘devices used to inhale a vapour, sometimes called vaping, which may contain nicotine and are commonly flavoured’. Images included mod-box and pen-style e-cigarettes. For awareness of e-cigarettes, pupils were asked ‘Had you heard of e-cigarettes before today?’ (response options: ‘Yes’, ‘No’ and ‘I don’t know’). For e-cigarettes use, children were asked ‘Have you ever used an e-cigarette’ (response options: ‘Yes, once’, ‘Yes, more than once’ and ‘No’). For smoking, pupils were asked (in all surveys) ‘Have you ever smoked tobacco?’ (response options: ‘Yes’, ‘No’). A single indicator of perceived smoking susceptibility asked children ‘Do you think that in two years’ time you will smoke?’, with response options: ‘Definitely yes, ‘Probably Yes’, ‘Maybe, maybe not’, ‘Probably not’ and ‘Definitely not’. Children giving any response other than ‘definitely not’ were classed as susceptible to smoking.

#### Parental smoking and vaping and exposure to smoke and e-cigarettes in the home and car

Pupils were asked ‘Do any of the following people smoke?’, i) father, ii) mother, iii) mother’s partner, iv) father’s partner, with a binary variable created indicating whether or not at least one of these parent figures smoked. This was repeated for e-cigarettes. Pupils were asked ‘How often do the following people smoke inside your home?’, i) father, ii) mother, iii) mother’s partner, iv) father’s partner, v) Other people you live with (e.g. aunt, uncle, parent’s friends, lodgers), vi) best friend, vii) siblings, viii) grandparents, ix) other people who come to our home (visitors). Response options for each person included: ‘smokes in the home every day’, ‘sometimes smokes in the home’, ‘does not smoke in the home’, ‘I don’t know’, ‘Don’t have or see this person’. These were combined into three binary variables relating to smoking in the home (either every day or sometimes) by parent figures, visitors and by anyone. Pupils were asked ‘While you were inside your home yesterday was anyone smoking there?’ as well as ‘Is smoking allowed inside your home?’, with response options of ‘no, smoking is not allowed at all’ (full restriction), ‘smoking is allowed in certain areas only’, ‘smoking is allowed only on special occasions in our home’ (partial smoking restrictions), ‘smoking is allowed anywhere in our home’ (no restriction), and ‘I don’t know’. For exposure to e-cigarettes in the home, pupils were asked, ‘Does anyone use an e-cigarette in a house while you are in it?’ with response options ‘yes, occasionally’, ‘yes, more than once a week’ (both coded as ‘yes’) and ‘no’. In 2014 and 2019, pupils were asked ‘How often are you in a car, van or truck where people are smoking?’, with response options ‘about every day’, ‘sometimes’, ‘never’, ‘I don’t know’. Response options ‘about every day’, ‘sometimes’ were combined to indicate being in a vehicle where people smoke at least sometimes. This was repeated for e-cigarette use. Whether smoking was allowed in the car was assessed by asking, “Are people allowed to smoke in your car, van or truck?’ with the response options: ‘Yes’, ‘No’, ‘I don’t know’ and “don’t have a family car, van or truck’, with children saying yes compared against all other responses. Exposure to smoke in a car the previous day was assessed by asking ‘While you were inside a car yesterday was anyone smoking there?’ with the response options ‘I wasn’t in car yesterday’, ‘there was no-one smoking there’, ‘yes, someone was smoking there’ and ‘I don’t know’, with children saying yes compared against all other responses.

#### Exposure to smoke and e-cigarette use in public places

In 2014 and 2019 pupils were asked, ‘In the last month, how often have you seen people smoking outside of or near the entrance to the following places’: Leisure facilities (for example swimming pools and sports clubs); The doctor’s surgery (a common UK term for the doctor’s office/practice); The hospital; Bus stations; Train stations; Cinema. Response options were i) Regularly, ii) Occasionally, iii) Never, iv) Haven’t been to this place in the last month. This was repeated for e-cigarette use. For each question separately, response options ‘regularly’ and ‘occasionally’ were combined and summed across locations to indicate the number of locations pupils reported having seen tobacco and e-cigarette use (respectively) at least once in the past month.

#### Composite indicator of exposure to tobacco and e-cigarettes

A variable was created to indicate on a scale of 0 to 3, whether children reported exposure to smoke in the following three places: in public places, at home and/or in a car (with 0 indicating no exposure and 3 exposure in all locations). The same variable was also constructed for e-cigarettes.

#### Perceived norms for smoking and vaping

For perceived prevalence of smoking in Wales pupils were asked to indicate i) how many people of their age in Wales smoked, and ii) how many adults in Wales smoked. Response options were ‘nearly all’, ‘about three-quarters’, ‘about half’, ‘about a quarter’, ‘hardly any’ and ‘I don’t know’. For child smoking prevalence, children giving any response greater than hardly any were compared against other responses. For adult smoking prevalence, children who gave a response option of more than half (i.e. ‘about three quarters’, or ‘nearly all’) were classed as perceiving that most adults smoke, and compared to all other responses. Pupils were also asked on a Likert scale ‘How do you feel when people smoke around you, for example in the same room or car?’, with response options ‘I mind a lot’, ‘I mind a bit’, ‘ I don’t mind very much’ and ‘I don’t mind at all’. Those who reported that they minded (a bit or a lot) were compared to those who didn’t mind (very much or at all).

### Statistical analyses

Frequencies and percentages were estimated overall for all outcomes. Odds ratios and 95% confidence intervals are reported from logistic regression analyses with year of survey entered as a categorical variable, with 2014 as the reference category. Models controlled for gender (with pupils who identified in 2019 as “prefer not to self-describe” or “prefer not to say” excluded due to the focus being change over time) and region with standard errors adjusting for clustering at the school level. We explored the inclusion of FAS in all models, however changes over time with these items (in particular the proliferation of computer ownership between 2008 and 2014), presented artificial increases between time points. The inclusion of an adjusted FAS score impacted results only where items for which there had been major social changes (i.e. computer ownership) and were not removed or standardised by year (see supplementary material). Hence, in all presented results, odd ratios are reported without adjustment for FAS.

For testing changes over time regarding smoking rules in the home, multinomial logistic regression models were used. Ordinal logistic regression was used for testing changes in exposure to tobacco and e-cigarettes outside public places.^1^ For all outcomes, models were applied to the whole sample and to a sub-group of pupils who reported that a parental figure smoked. Finally, to assess associations of exposure to tobacco and e-cigarettes with perceived smoking norms, indices for tobacco and e-cigarette were modelled individually and combined, with mutual adjustment for parental smoking and vaping status, as well as pupil e-cigarette use and smoking status, time, socioeconomic status, region and gender. Consistent with earlier analyses of change over time using CHETS survey, complete case analysis was used. While missingness was trivial for most individual items, when combining many items in models this increased. This is indicated as a footnote in the results tables where applicable. Weights were not applied to the analyses as the sample showed no substantial departure from population estimates of socioeconomic status (i.e. Free School Meal entitlement) and regional distribution of pupils across Wales. All analyses were carried out using Stata 14.0.

## Results

### School survey sociodemographic information

Response rates for individual surveys are reported elsewhere (31, 32). While approximately two-thirds of schools approached participated in surveys from 2007 to 2014, in 2019, school level response rates declined to 39%. Response rates for pupils within schools remained at approximately 90% for each survey round. Sociodemographic characteristics are presented in Table 1. Distributions of sociodemographic variables were similar across time, except for a large increase in pupils reporting ownership of two or more computers (used within the FAS scale) from 2008 to 2014.

**Table 1:** Sociodemographic characteristics across CHETS survey samples

	2007 *n* = 1546	2008 *n* = 1568	2014 *n* = 1474	2019 *n* = 2153
Gender^a^				
Male	750 (48.5%)	773 (49.3%)	734 (49.8%)	1103 (51.2%)
Female	796 (51.5%)	795 (50.7%)	740 (50.2%)	1050 (48.8%)
Lives with^b^				
Both parents	1065 (68.9%)	1062 (67.7%)	987 (67.0%)	1474 (68.5%)
Step family	165 (10.7%)	170 (10.8%)	142 (9.6%)	199 (9.2%)
Single mum	259 (16.8%)	268 (17.1%)	261 (17.7%)	344 (16.0%)
Single dad	18 (1.2%)	23 (1.5%)	31 (2.1%)	29 (1.4%)
Grandparents	17 (1.1%)	19 (1.2%)	22 (1.5%)	28 (1.3%)
Care/foster home	4 (0.3%)	8 (0.5%)	11 (0.8%)	12 (0.6%)
Other/missing	18 (1.2%)	18 (1.2)	20 (1.4%)	67 (3.1%)
Wales Region				
North	345 (22.2%)	346 (22.1%)	289 (19.6%)	441 (20.5%)
South	1013 (65.5%)	1014 (64.7%)	967 (65.6%)	1472 (68.4%)
West	64 (4.1%)	81 (5.2%)	42 (2.9%)	98 (4.6%)
Mid	124 (8.0%)	127 (8.1%)	176 (11.9%)	142 (6.6%)
Family affluence				
Child has their own bedroom	1209 (78.1%)	1217 (78.2%)	1132 (77.3%)	1639 (76.6%)
Family has a car or van				
No	115 (7.5%)	115 (7.4%)	108 (7.4%)	152 (7.1%)
Yes, one	614 (40.0%)	557 (35.8%)	618 (42.2%)	792 (37.0%)
Yes, two	808 (52.6%)	886 (56.9%)	737 (50.4%)	1196 (55.9%)
Family owns a computer				
None	54 (3.5%)	66 (4.2%)	20 (1.4%)	40 (1.9%)
One	641 (41.7%)	562 (36.0%)	120 (8.2%)	178 (8.3%)
Two	494 (32.1%)	501 (32.1%)	225 (15.4%)	332 (15.5%)
More than two	348 (22.6%)	434 (27.8%)	1099 (75.1%)	1592 (74.3%)

^a^Pupils who identified as “prefer not to self-describe” or “prefer not to say” were excluded due to the focus being change over time ^b^Due to previous heteronormativity of response options, additional items were added in 2019 to include same-sex parents and are reported here as “both parents”

### Smoking susceptibility, smoking experimentation, e-cigarette awareness and e-cigarette experimentation

**Fig. 1:**
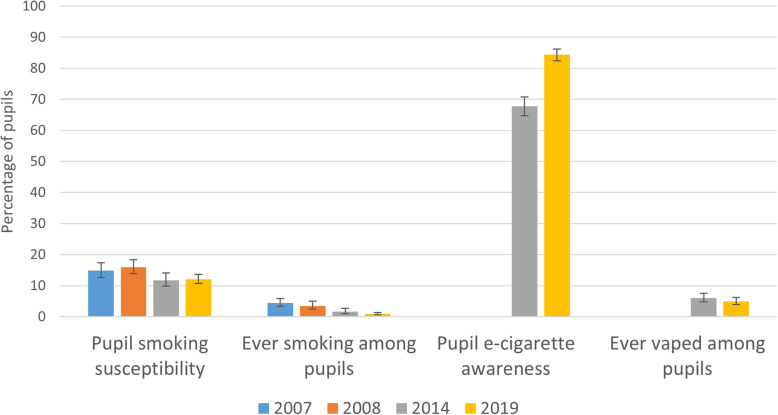
Change in pupil smoking susceptibility, smoking, e-cigarette awareness and vaping

As indicated in Table 2, for smoking susceptibility, odds ratios contrasting both 2008 and 2007 with 2014 indicate significantly higher susceptibility to smoking in earlier datapoints (i.e. reduced susceptibility over time). There was however no evidence of further decline in susceptibility from 2014 to 2019 (see also Fig. 1). While odds ratios for a sub-sample limited to children with at least one parent figure who smoked followed a similar pattern, changes over time were not significant. For the whole sample and for children of smokers, the odds of having ever smoked were greater in 2007 and 2008 relative to 2014, with a graded reduction in the odds of ever smoking over time. For both groups, odds ratios for contrasts between 2014 and 2019 were in the direction of continued lowering of the odds of ever smoking, though were of borderline significance for the whole sample only. The odds of having heard of e-cigarettes increased significantly from 2014 to 2019 for the whole sample, and for children of smokers. There was no evidence of change in odds of having tried an e-cigarette for the whole sample, or among children of smokers.

**Table 2:** Change in smoking norm perceptions as well as pupil use, and exposure to, smoking and e-cigarettes with ORs and 95% CI for logistic regression analyses (unless otherwise stated) adjusted for gender and region

		Whole sample		Children with at least one smoking parent figure	
		OR (95% CI)	***p***	OR (95% CI)	***P***
Pupil’s awareness of e-cigarettes, ever use of e-cigarettes and tobacco, and smoking susceptibility					
Smoking susceptibility *N* = 6647/2654	2007	1.32 (1.01 to 1.72)	0.045	1.18 (0.80 to 1.73)	0.407
	2008	1.43 (1.10 to 1.87)	0.008	1.32 (0.90 to 1.91)	0.151
	2014	1.00	–	1.00	–
	2019	1.03 (0.80 to 1.32)	0.838	0.95 (0.67 to 1.35)	0.782
Ever smoked *N* = 6711/2677	2007	2.85 (1.68 to 4.82)	< 0.001	2.99 (1.55 to 5.76)	0.001
	2008	2.20 (1.17 to 4.12)	0.015	2.89 (1.37 to 6.10)	< 0.001
	2014	1.00	–	1.00	
	2019	0.53 (0.27 to 1.04)	0.066	0.71 (0.33 to 1.56)	0.396
Heard of e-cigarettes *N* = 3533/1278	2014	1.00		1.00	
	2019	2.56 (2.12 to 3.10)	< 0.001	3.45 (2.56 to 4.65)	< 0.001
Ever used an e-cigarette *N* = 3499/1268	2014	1.00	–	1.00	
	2019	0.80 (0.57 to 1.13)	0.206	0.95 (0.59 to 1.50)	0.812
Parental smoking and vaping					
Parent figures smoke *N* = 6475	2007	1.32 (1.10 to 1.58)	0.003	–	
	2008	1.18 (0.99 to 1.39)	0.060	–	
	2014	1.00	–	–	
	2019	0.85 (0.68 to 1.05)	0.130	–	
Parent figure uses e-cigarettes *N* = 3330^b^/1203	2014	1.00	–	1.00	–
	2019	1.26 (1.00 to 1.57)	0.046	1.24 (0.97 to 1.57)	0.085
Exposure to tobacco and e-cigarettes in the home					
Parent figures smoke in the home *N* = 6501/2605	2007	2.05 (1.71 to 2.47)	< 0.001	2.71 (2.05 to 3.57)	< 0.001
	2008	1.73 (1.43 to 2.10)	< 0.001	2.21 (1.71 to 2.84)	< 0.001
	2014	1.00	–	1.00	–
	2019	0.79 (0.63 to 0.99)	0.042	0.80 (0.64 to 1.00)	0.046
Someone smoking in home yesterday *N* = 6605/2626	2007	2.35 (1.88 to 2.94)	< 0.001	2.58 (2.03 to 3.27)	< 0.001
	2008	2.21 (1.78 to 2.74)	< 0.001	2.49 (1.98 to 3.13)	< 0.001
	2014	1.00	–	1.00	–
	2019	0.88 (0.69 to 1.11)	0.273	1.01 (0.79 to 1.29)	0.957
Smoking rules in the home^a^ *N* = 5915/2369	Partial vs full restriction. Full restriction is reference				
	2007	1.52 (1.26 to 1.83)	< 0.001	1.56 (1.24 to 1.95)	< 0.001
	2008	1.39 (1.15 to 1.67)	0.001	1.71 (1.37 to 2.13)	< 0.001
	2014	1.00	–	1.00	–
	2019	1.13 (0.91 to 1.40)	0.262	1.40 (1.11 to 1.76)	0.005
	None vs full restriction. Full restriction is reference				
	2007	4.33 (2.91 to 6.44)	< 0.001	4.63 (3.00 to 7.14)	< 0.001
	2008	3.36 (2.27 to 4.98)	< 0.001	4.16 (2.73 to 6.32)	< 0.001
	2014	1.00	–	1.00	–
	2019	1.21 (0.83 to 1.77)	0.324	1.23 (0.81 to 1.89)	0.333
Visitors smoke in the home *N* = 5536/2032^b^	2007	2.46 (2.01 to 3.01)	< 0.001	2.83 (2.19 to 3.65)	< 0.001
	2008	1.89 (1.54 to 2.32)	< 0.001	2.46 (1.87 to 3.24)	< 0.001
	2014	1.00	–	1.00	–
	2019	0.66 (0.52 to 0.82)	< 0.001	0.72 (0.54 to 0.95)	0.023
People use e-cigarettes in the home *N* = 3477/1256	2014	1.00	–	1.00	–
	2019	1.41 (1.13 to 1.77)	0.003	1.48 (1.12 to 1.95)	0.006
Exposure to tobacco and e-cigarettes in a car					
Smoking allowed in family car *N* = 6689/2669	2007	2.55 (2.07 to 3.15)	< 0.001	2.41 (1.90 to 3.05)	< 0.001
	2008	2.20 (1.75 to 2.76)	< 0.001	2.13 (1.70 to 2.68)	< 0.001
	2014	1.00	–	1.00	–
	2019	0.67 (0.52 to 0.88)	0.004	0.65 (0.50 to 0.85)	0.002
In a car where someone was smoking yesterday *N* = 6615/2632	2007	1.83 (1.31 to 2.56)	0.001	1.90 (1.30 to 2.78)	0.001
	2008	1.79 (1.31 to 2.43)	< 0.001	1.90 (1.35 to 2.67)	< 0.001
	2014	1.00	–	1.00	–
	2019	0.52 (0.36 to 0.75)	0.001	0.57 (0.37 to 0.88)	0.012
Sometimes in a car where people are smoking *N* = 3587/1305	2014	1.00		1.00	
	2019	0.64 (0.52 to 0.78)	< 0.001	0.54 (0.42 to 0.70)	< 0.001
Someone uses e-cigarettes while I am inside car *N* = 3463/1249	2014	1.00	–	1.00	–
	2019	1.56 (1.19 to 2.05)	0.002	1.76 (1.25 to 2.48)	0.001
Exposure to smoking and e-cigarettes in public places^c^					
Number of public places children reported seeing smoking in the past month *N* = 3544 / 1284	2014	1.00	–	1.00	–
	2019	0.74 (0.64 to 0.86)	< 0.001	0.6 (0.48 to 0.76)	< 0.001
Number of public places children reported seeing e-cigarettes in the past month *N* = 3461/1260	2014	1.00	–	1.00	–
	2019	2.18 (0.95 to 1.26)	< 0.001	1.80 (1.43 to 2.26)	< 0.001
Perceived smoking norms					
Most adults in Wales smoke *N* = 6672/2665	2007	1.39 (1.15 to 1.68)	0.001	1.09 (0.85 to 1.40)	0.483
	2008	1.23 (1.02 to 1.48)	0.030	1.11 (0.85 to 1.45)	0.461
	2014	1.00	–	1.00	–
	2019	0.66 (0.54 to 0.80)	< 0.001	0.65 (0.50 to 0.84)	0.001
More than hardly any children my age smoke *N* = 6631/2645	2007	1.54 (1.24 to 1.90)	< 0.001	1.28 (0.97 to 1.68)	0.078
	2008	1.36 (1.10 to 1.67)	0.005	1.20 (0.90 to 1.60)	0.203
	2014	1.00	–	1.00	–
	2019	0.69 (0.55 to 0.86)	0.001	0.68 (0.51 to 0.91)	0.010
Mind people smoking around me *N* = 6591/2631	2007	1.28 (1.00 to 1.65)	0.052	1.58 (1.15 to 2.18)	0.005
	2008	1.25 (0.99 to 1.59)	0.064	1.40 (1.03 to 1.90)	0.032
	2014	1.00	–	1.00	–
	2019	0.68 (0.53 to 0.88)	0.003	0.88 (0.63 to 1.22)	0.428

^a^ORs and 95% CI’s from multinomial logistic regression analyses ^b^missing data exceeds 5% for this item; ^c^ ORs and 95% CI’s from ordinal logistic regression analyses

### Parental smoking and use of e-cigarettes

**Fig. 2:**
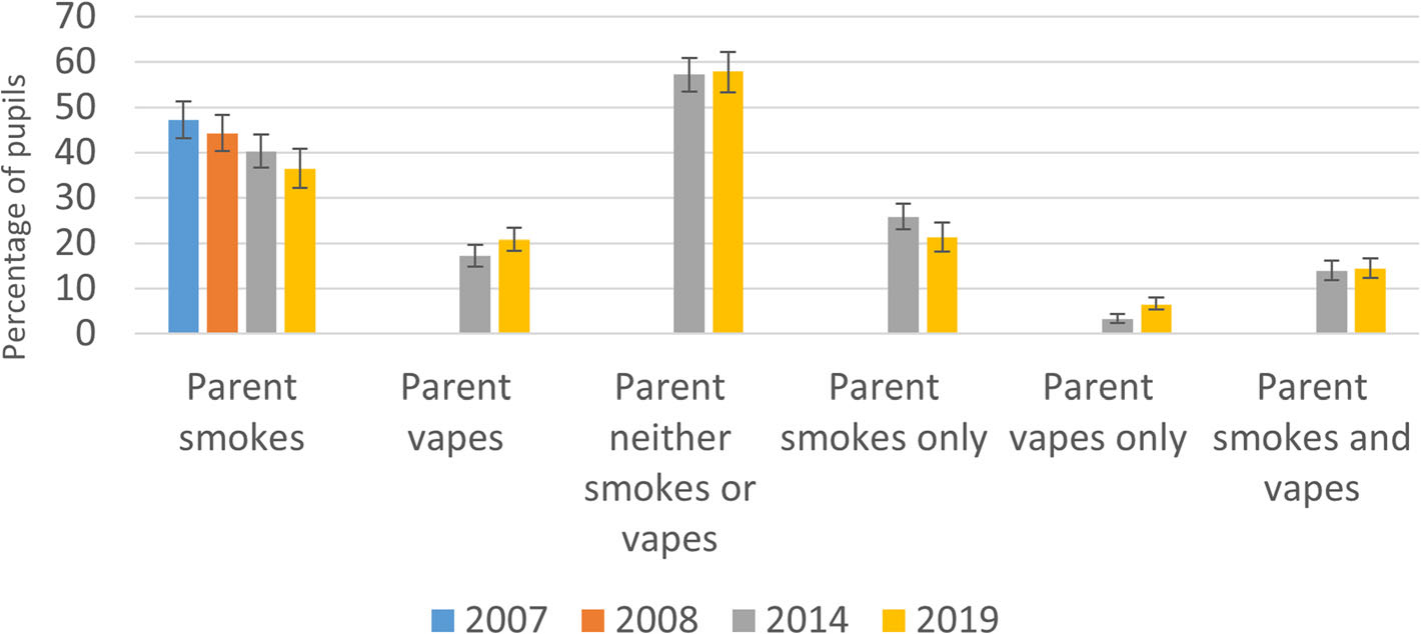
Change in parental smoking, vaping and dual use

Odds ratios for whether children reported that at least one parent figure smoked indicated a graded trend over time, with levels highest at the start of the time series and declining from 2014 to 2019, although only the contrast between 2014 and 2007 was significant. The percentage of children reporting that a parent figure used e-cigarettes increased significantly from 2014 to 2019, with a similar odds ratio but falling short of significance for the subsample of children of smokers. For dual use of tobacco and e-cigarettes, this remained similar in 2014 (13.9%) and 2019 (14.4%; Fig. 2 and Table S3). However, there was a change in the single use of each product. The percentage with parent figures who smoked but no parent figure who vaped decreased from 25.7% in 2014 to 21.2% in 2019, while the percentage with parent figures who used e-cigarettes only and did not smoke increased from 3.2% in 2014 to 6.5% in 2019 (Table S3).

### Exposure to tobacco and e-cigarettes in the home

**Fig. 3:**
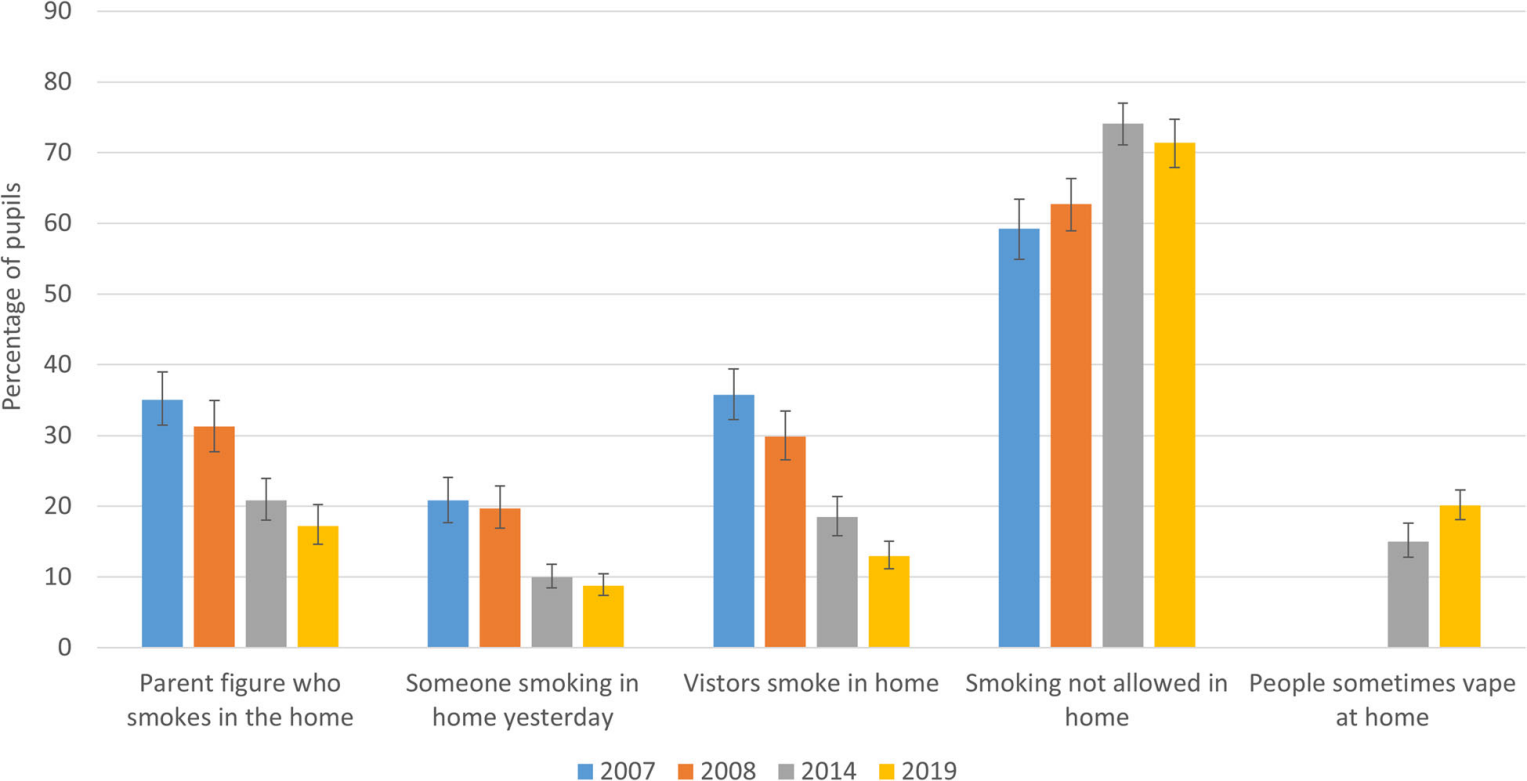
Change in vaping and smoking in the home

For whether children reported that parent figures smoked ‘in the home’, a graded relationship was observed, with all contrasts significant, indicating a continued reduction in the percentage of children reporting that parent figures smoked in the home (Table 2, Fig. 3). This pattern was evident when limited to children of smokers, and hence was unlikely to be an artefact of systematic changes in the likelihood of reporting that parent figures smoked. For the percentage of children reporting that someone was smoking in their home yesterday, again, odds ratios indicated a graded decline over the time series, though with contrasts between 2014 and 2019 not reaching significance, and no change from 2014 to 2019 evident for children of smokers. The odds of reporting partial restrictions on smoking in the home and the odds of reporting no restrictions (both relative to full restriction) were significantly higher in 2007 and 2008 than in 2014, indicating a graded decline, although there was no further evidence of decline from 2014 to 2019, and some evidence of increased odds of partial restriction among children of smokers. For whether children reported that visitors who came into their home smoked, there was a graded relationship indicating a continued decline over time, in the whole sample and among children of smokers. Among the whole sample, and children of smokers, the odds of reporting that e-cigarettes were used inside the home increased significantly from 2014 to 2019.

**Fig. 4:**
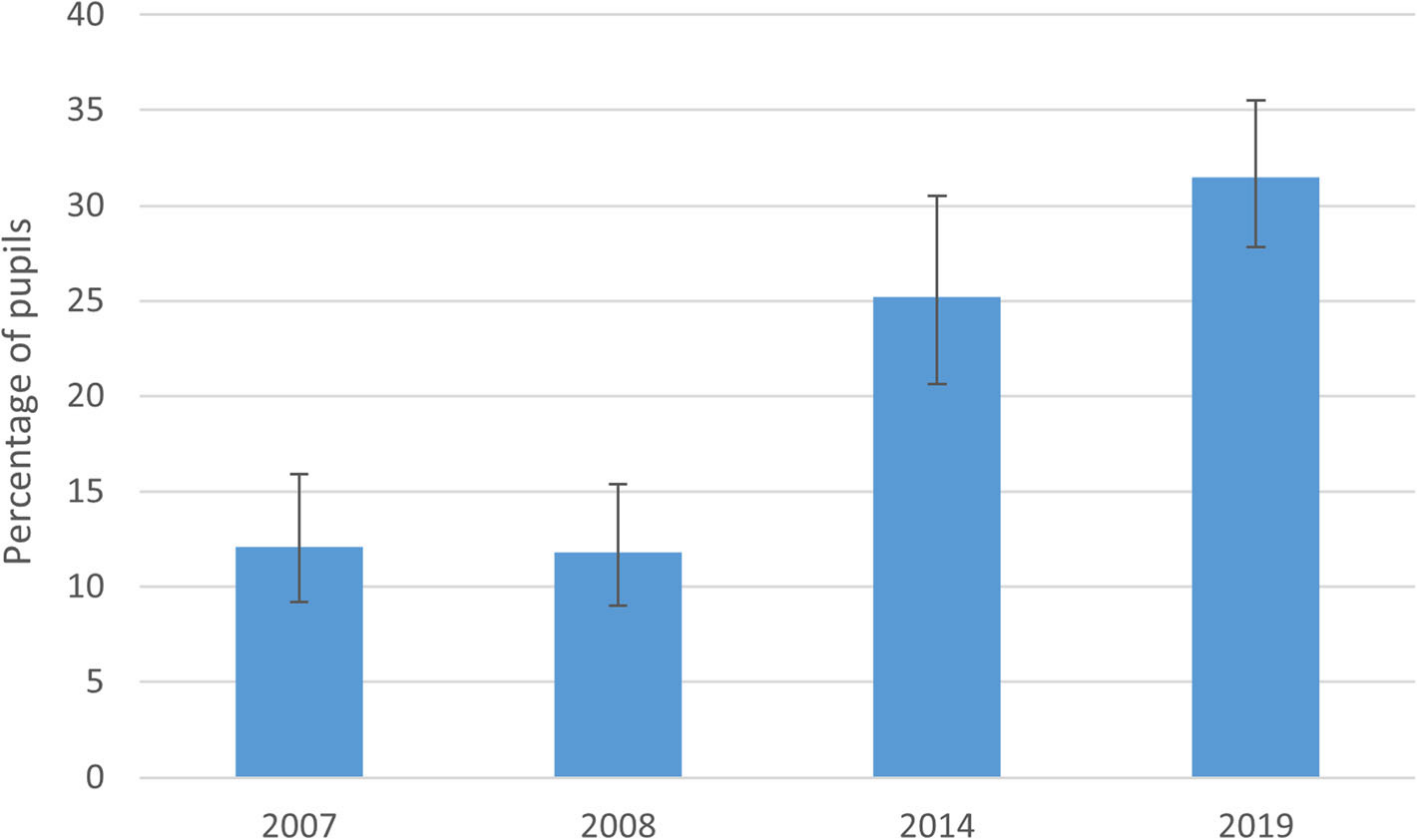
Change in the percentage of pupils reporting that smoking is *not prohibited* in their home, but identifying *no-one who does* smoke in the home

To assess whether the apparent inconsistency of the item on smoking rules in the home with other measures of exposure may be due to the changing relevance of the question over time (i.e. formal rules may be less necessary where there is a tacit assumption that something will not happen), as a post-hoc analysis, we examined changes in the percentage of pupils who reported that smoking was not prohibited in the home, but identified no-one who did smoke in the home (Fig. 4). Substantial change emerged over time, with 31.5% of children who did not report smoking restrictions in their home in 2019 nevertheless not identifying anyone who did smoke in their home, compared to 12.1% in 2007.

### Change in exposure to tobacco and e-cigarettes in cars

**Fig. 5:**
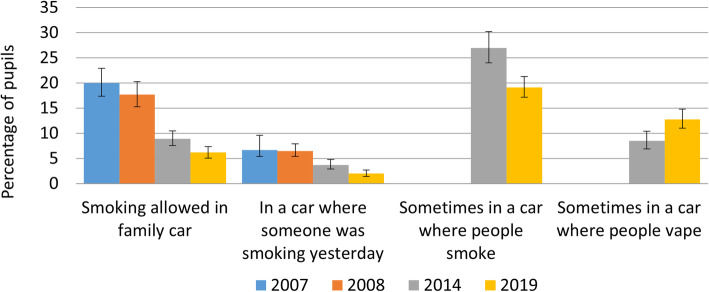
Change in smoking and vaping in cars

Across all indicators of exposure to smoking in cars, odds ratios indicated that the odds of exposure were higher in 2007 and 2008 than in 2014, and that odds continued to decline significantly from 2014 to 2019, with all contrasts significant for the whole sample and among children of smokers (Table 2, Fig. 5). There was also significant evidence of growth in reported exposure to e-cigarettes in cars, in the whole sample, and among children of smokers.

### Change in exposure to smoking and e-cigarettes in public places

**Fig. 6:**
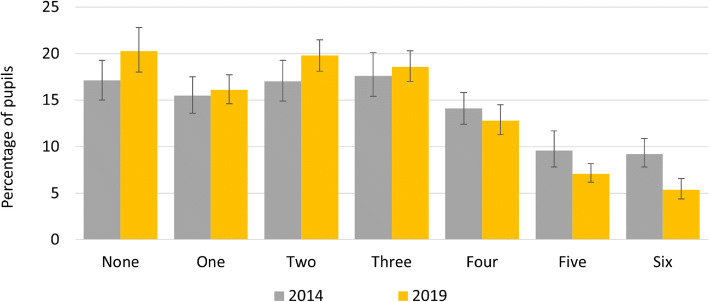
Change in the number of public places pupils saw smoking in the past month

**Fig. 7:**
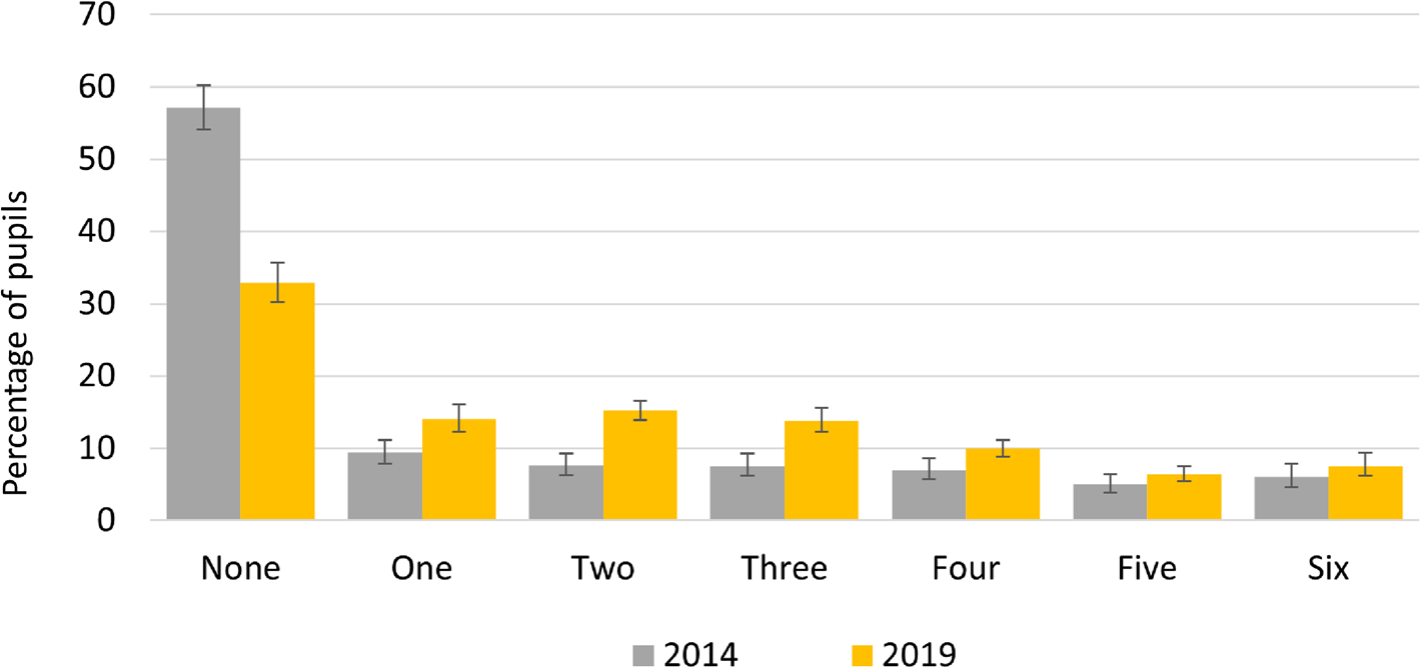
Change in the number of public places pupils saw vaping in the past month

Reported exposure to smoking in public places decreased significantly from 2014 to 2019 (Table 2, Fig. 6); while exposure to e-cigarettes in the same public places increased (Fig. 7). Exposure to smoking remained more prevalent than exposure to e-cigarettes. Between 2014 to 2019 pupils with a smoking parent figure reported seeing smoking in significantly fewer public places (Table 2). During this time both groups reported an increase in seeing more vaping in public places; however, this change was greater among pupils with no smoking parent figure (Table 2).

### Change in perceived smoking norms

**Fig. 8:**
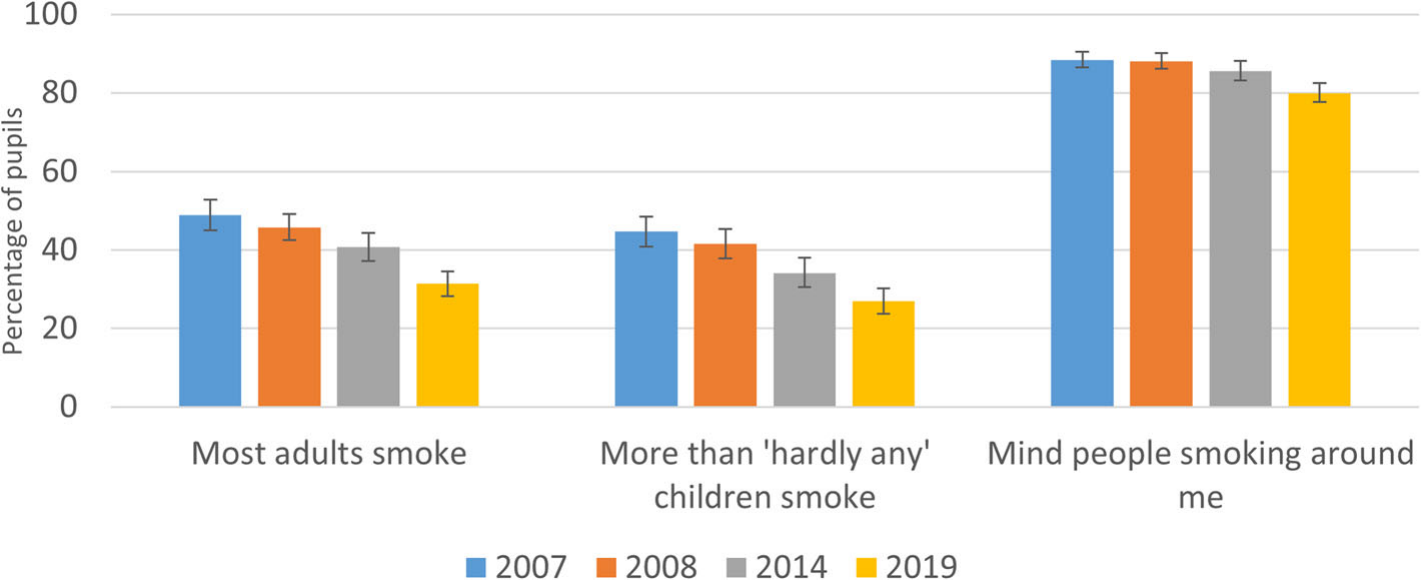
Change in perceived smoking norms

Among the whole sample, the odds of reporting a perception that most adults smoke was greater in 2007 and 2008 than in 2014 and continued to decline significantly from 2014 to 2019 indicating a graded decline over time, (Table 2, Fig. 8). A somewhat different pattern was however evident for children of smokers, among whom there was limited evidence of decline in smoking norms from 2007 through to 2014, although the odds of perceiving that most adults smoked declined substantially from 2014 to 2019. These same patterns were replicated for the odds of perceiving that more than hardly any children smoke, with decline from 2014 to 2019 similar in the whole sample and among children of smokers, though with less clear evidence of a decline in smoking norms from 2007 to 2014 among children of smokers. For whether young people reported that they mind others smoking around them however, there was evidence of a graded relationships indicative of lower objection to (i.e. greater tolerance) of others’ smoking over time, with contrasts of 2007 and 2008 with 2014 marginally significant, and further decline evident from 2014 to 2019, although smaller and not significant for children of smokers.

### Associations of exposure to tobacco and e-cigarettes with perceived smoking norms

Exposure to a greater number of locations where pupils reported seeing tobacco (i.e. in a public place, at home or in a car) was strongly associated with higher odds of perceiving that most adults smoke (*p* < 0.001), that more than ‘hardly any’ children smoke (*p* < 0.001), and not minding people smoking around them (*p* < 0.001) (Table 3). Exposure to a greater number of locations where pupils reporting seeing e-cigarettes (i.e. in a public place, at home or in a car) was also associated with higher odds of perceiving that most adults smoke (*p* < 0.001), perceiving that more than ‘hardly any’ children smoke (*p* < 0.001) and not minding people smoking around them (*p* < 0.001). When mutually adjusted for both tobacco and e-cigarette exposure, exposure to tobacco smoke retained a significant and graded association with all items (see Table 3). Associations of exposure to e-cigarettes with normative items were attenuated with a less clear relationship with feelings about people smoking around them and perceptions that most adults smoke. Exposure to e-cigarettes retained a significant association with perceiving that more than ‘hardly any’ children smoke in mutually adjusted models; however, models in Table 3 should be interpreted with caution due to levels of missing data.

**Table 3:** Odds ratios and 95% confidence intervals from multivariable logistic regression analyses of associations between exposure to tobacco and e-cigarettes and perceived smoking norms

		Most adults smoke (*n* = 3008)		More than hardly any children smoke (*N* = 2956)		Mind people smoking around me (*n* = 2982)	
		OR (95% CI)	***p***	OR (95% CI)	***p***	OR (95% CI)	***p***
Parent figures smoke		1.92 (1.61 to 2.30)	< 0.001	1.13 (0.94 to 1.36)	0.209	0.58 (0.44 to 0.77)	< 0.001
Parent figures use e-cigarettes		1.15 (0.91 to 1.45)	0.249	1.00 (0.77 to 1.29)	0.979	0.94 (0.70 to 1.25)	0.661
The number of locations exposed to tobacco use	0	1.00	–	1.00	–	1.00	–
	1	1.39 (1.07 to 1.80)	0.015	1.20 (0.92 to 1.55)	0.172	0.86 (0.62 to 1.18)	0.350
	2	2.09 (1.55 to 2.81)	< 0.001	1.57 (1.17 to 2.09)	0.003	0.59 (0.43 to 0.81)	0.001
	3	2.71 (1.91 to 3.86)	< 0.001	2.47 (1.70 to 3.57)	< 0.001	0.39 (0.26 to 0.58)	< 0.001
	Wald chi-square	16.07	< 0.001	9.74	< 0.001	9.18	< 0.001
The number of locations exposed to e-cigarettes	0	1.00	**–**	1.00	–	1.00	–
	1	1.16 (0.92 to 1.46)	0.119	1.28 (1.05 to 1.55)	0.013	1.19 (0.89 to 1.60)	0.241
	2	1.04 (0.74 to 1.46)	0.806	1.62 (1.11 to 2.36)	0.013	0.74 (0.53 to 1.02)	0.067
	3	1.24 (0.85 to 1.80)	0.256	1.44 (0.93 to 2.24)	0.101	0.86 (0.54 to 1.39)	0.547
	Wald chi-square	0.84	0.474	3.19	0.026	1.90	0.134

Models adjust for gender, FAS (family affluence scale), region, time, pupil smoking and e-cigarette use. Values 1, 2 and 3 represent the composite indicators of exposure to tobacco and e-cigarettes compared to the reference category of ‘0’

## Discussion

Our findings indicate that childhood experimentation with tobacco among 10–11 year olds in Wales decreased from 5 to 1% from 2007 to 2019, and has almost been eliminated following a period of increasingly comprehensive tobacco control policy (3, 4). In this time period, e-cigarettes have emerged in the UK (3, 4, 18, 20). By 2014, ever use of e-cigarettes among pupils was comparable to ever use of tobacco in 2007. However, while pupil awareness of e-cigarettes grew rapidly from 2014 to 2019, ever e-cigarette use has neither decreased nor increased significantly. Notably, adolescent data from Wales reported elsewhere shows that while experimentation with vaping increased from 2013 to 2017, it had declined again by 2019 (45). It is unclear whether vaping experimentation among children remained steady from 2014 to 2019 or has also risen and decreased within this period. Child intentions for future smoking, while declining from 2008 to 2014, have however remained stable since 2014.

Child reports of smoking by their parent figures declined significantly since 2007. As parental smoking represents an important mechanism in the intergenerational transmission of smoking (46), reduced parental smoking has likely played an important role in driving aforementioned reductions in childhood experimentation. By contrast, child reports of parental e-cigarette use rose significantly from 2014 to 2019. Most children who reported that a parent vapes also reported parental smoking at both time-points. However, reports that parent figures used *only* e-cigarettes increased over time, matched by a decrease in those who reported that parents *only* smoked. While data are not longitudinal, this shift may indicate that a growing proportion of parents in Wales are moving fully from smoking to vaping, consistent with data from elsewhere finding that e-cigarettes have become the most commonly used smoking cessation device in Wales (47).

By most measures, children’s exposure to secondhand smoke in homes, cars and public places declined across the time-series, and continued to decline from 2014 to 2019. There was greater consistency across measures for continued reductions of smoking in cars than in homes since 2014, which might reflect a role of 2015 legislation prohibiting smoking in cars in maintaining downward pressure on trends for smoking in cars, which had decreased rapidly to that point (48). The main exception was the percentage of children reporting family rules restricting smoking in the home, which after increasing from 2007 to 2014, showed no further change since 2014. However, in our qualitative research (33), children talked of not having restrictions in their home because it was something that just did not happen, and was understood to be unacceptable without a need for formal rules. Hence, questions about smoking rules in the home may be losing their validity as indicators of whether smoking happens in the home as smoking in children’s home becomes more de-normalised. Consistent with this interpretation, a post-hoc analysis informed by our qualitative research indicated that the percentage of children who reported no rule on smoking in the home, but nevertheless, reported that nobody did smoke in their home increased substantially across the time-series. Notably however, there was also only a small and non-significant reduction from 2014 to 2019 in the percentage of children reporting that someone was smoking in their home the previous day. Hence, where indicators pointed to continued significant change, this was in a direction of continued decline, but there was more mixed evidence for change in exposure to smoke in homes relative to other locations.

In contrast with trends for tobacco exposure, exposure to e-cigarettes increased in all locations. The extent to which childhood exposure to e-cigarettes represents a public health problem regardless of links to smoking, remains a source of debate. In the US, some have expressed concern surrounding the potential harm for bystanders of secondhand vapour (45–47). While less harmful than secondhand tobacco smoke, some argue that it is not entirely without risk (49, 50) . Some have argued that a precautionary approach should be taken, with parents educated about potential harms (51) and e-cigarette users offered NRT to protect children from e-cigarette aerosols (52). In the UK, others have highlighted the need to assess nicotine exposure through vaping in cars to inform policies that might protect children (53). However, others argue that e-cigarettes offer opportunities to reduce childhood secondhand smoke exposure in environments where tobacco would otherwise be used (38). In both the US and UK, approaches to restricting e-cigarettes in private spaces (i.e. households) have been found to be less formalised and ‘rigid’ than for smoking cigarettes (23, 46, 51, 54). As most adult e-cigarette users are current or ex-smokers (18), a tendency for increased use of e-cigarette use in homes and cars perhaps reflects concern for protecting children from the harms of tobacco smoke, with e-cigarettes potentially representing one mechanism for keeping homes and cars free from tobacco smoke. However, further longitudinal research is needed to understand whether the growth of e-cigarette use reflects parents who would otherwise smoke in those locations now vaping instead, or introduces vaping into environments in which smoking had already been eliminated.

Growing visibility of e-cigarettes has led to debates over whether e-cigarettes may renormalise smoking, due to perceived similarities with traditional cigarettes (26, 55). Re-renormalisation concerns have been a major driver of policy efforts (56, 57), including the failed proposal to ban vaping in public places in Wales (28). However, similar to other recent studies among adolescents (11, 58), our findings of the continued decline in experimentation with and exposure to tobacco, in addition to continuing declines in perceived smoking norms for smoking, does not support the idea that renormalisation is occurring. Most normative measures showed continued decline, with fewer children in 2019 perceiving that most adults smoke, or that smoking was common in children their age. However, there was some evidence of diminishing disapproval of secondhand smoke over time since 2007, with fewer children saying they mind people smoking around them (although a large majority did say this at all timepoints). It is likely that children’s objection to people smoking around them peaked around the time of the high-profile introduction of smoke-free legislation, and associated campaign work. As fewer children experience exposure to secondhand smoke in their daily lives, they are perhaps less likely to hold a strong attitude toward hypothetical exposure. Notably, while disapproval of secondhand smoke remained lowest among those exposed to tobacco smoke (mirroring qualitative research that children who are exposed to it express a strong dislike of it (33)), recent change over time in tolerance of secondhand smoke was not observed among children of smokers, perhaps indicating that this change was driven primarily by children with relatively low exposure to secondhand smoke.

However, taken together with the tendency for the lack of increase in formal rules on smoking, greater acceptance by children of people smoking around them may reflect a tendency for more limited communication with children regarding tobacco, and a perception that smoking is not an issue which affects children anymore. In our linked qualitative research, many non-smoking parents expressed a view of smoking as an adult issue, and a preference not to discuss tobacco with their children until they began to encounter it later in adolescence (33). Anecdotally, during survey recruitment, some schools who declined to participate also expressed a view that tobacco was not really an important issue for children in their school anymore. Notably, while children’s experimentation with tobacco and perceived norms continued to decline, there was also little change in perceived susceptibility to future smoking from 2014 to 2019. Given that the most recent adolescent data in the UK show that following a long period of decline, adolescent tobacco use (along with other substances such as cannabis) is no longer decreasing (59), it remains important to understand children’s perceptions of tobacco and drivers of these perceptions from a young age.

There was a clear graded relationship of exposure to tobacco with all markers of perceived smoking norms. Children with higher exposure to tobacco were more likely to perceive smoking as a normal adult behaviour, something that children do, and not to mind others smoking around them. Exposure to e-cigarette use showed a similar graded relationship, where tobacco exposure was not adjusted for. However, after adjusting for the tendency of children with high exposure to e-cigarettes to also be more likely to have high exposure to tobacco, a less clear relationship of e-cigarette exposure with tobacco norms emerged. There was little evidence of a relationship of exposure to e-cigarette with perceptions of adult smoking as a normative behaviour, or children minding that people smoked around them, although consistent with (25), an association with perceiving that more than ‘hardly any’ children smoked remained. Hence, consistent with our previous analyses which found that associations of parental vaping with smoking norms were attenuated by parental smoking (34), these data are consistent with a conclusion that associations of exposure to e-cigarettes with smoking norms are largely explained by the tendency for children who have high exposure to e-cigarettes also to have high exposure to tobacco.

Our study benefits from using nationally representative surveys of pupils in Wales, repeated over a span of 12 years. However, limitations include a repeat cross-sectional survey design, which means that causation cannot be inferred. It also relies on self-report measures that are subject to biases which may have changed over time. Declines in parental smoking for example may indicate that parents are hiding their smoking from their children, or that children are increasingly reluctant to report that parents smoke, given increased stigmatisation. Further, in line with recent trends in social surveys, the 2019 survey achieved a substantially lower response rate than earlier rounds. While this did not impact representativeness according to measures obtained, estimates of change over time may be confounded by unmeasured differences in samples due to differential response biases. While for the vast majority of items, data completeness was very high, for some models missingness exceeded 5% and should therefore be treated with caution. Our sample consisted of Year 6 pupils (age 10–11) and findings may not generalise to older youth where smoking and e-cigarette rates tend to be higher (11, 42). Our measurement of smoking susceptibility was limited to one questionnaire item to enable change over time with past surveys; however this differs from other existing definitions using multiple items (60, 61). While for many items, survey design is bounded by decisions made in earlier surveys, with item consistency maintained to enable analysis of change over time, our analysis of the increasing disjuncture between reports of home smoking rules and actual smoking in the home illustrates that question meanings may change over time, posing challenges for interpreting these as evidence of change over time in the underlying construct, or in how they are interpreted in an environment of diminished smoking prevalence.

## Conclusions

Notwithstanding the above caveats, this study provides evidence that within primary schools in Wales, children’s experimentation with, and exposure to, tobacco cigarettes continues to decline, representing a major success of comprehensive tobacco control measures in recent years. There is consistent evidence of declines in exposure to secondhand smoke in cars, with slightly less consistent evidence that the earlier declines to exposure in the home from 2007 to 2014 have continued post 2014. These declines appear to have largely continued alongside co-occurring increases in exposure to e-cigarettes. Ongoing surveillance of trends in childhood perceptions of tobacco and e-cigarettes remains a priority as the products and the landscape in which they are bought and sold continue to evolve. Longitudinal research is needed to better understand patterns in parental movement from tobacco cigarettes to e-cigarettes, including whether e-cigarettes are displacing tobacco smoke in homes and cars, or are being introduced to environments where tobacco smoking had already been eliminated. While these findings indicate that substantially fewer children are now exposed to tobacco, experiment with tobacco themselves, or view tobacco as a normal adult behaviour than in 2007, a sizeable minority continue to be exposed to adults’ tobacco use, and to misperceive that most adults smoke. If further declines in uptake of smoking in youth and further reductions in children’s exposure to tobacco are to be achieved, it is important not to be complacent and that childhood perceptions of, and exposure to, tobacco continue to be prioritised for public health intervention.

## Supplementary Information

**Additional file 1.**

## Data Availability

The datasets used and/or analysed during the current study are available from the corresponding author on reasonable request.

## Abbreviations

NRT: Nicotine Replacement Therapy
CHETS: Child Exposure to Environmental Tobacco Smoke
FAS: Family affluence scale

## Footnotes

1 Testing proportional odds assumptions revealed violation for e-cigarettes, but not smoking; thus ordinal models are reported with binary models reported in the supplementary file.
