# Supplementary material for: Changes in childhood experimentation with, and exposure to, tobacco and e-cigarettes and perceived smoking norms: a repeated cross-sectional study of 10–11 year olds’ in Wales
Source: BMC Public Health. 2021 Oct 23;21:1924. doi: 10.1186/s12889-021-12004-z (PMC8542319; doi:10.1186/s12889-021-12004-z)
Supplement: Supplementary file 1 — Additional file 1. [file 12889_2021_12004_MOESM1_ESM.docx]

**SUPPLEMENTARY INFORMATION**

**Statistical Analysis**

For comparisons between 2014 and 2019, we adjusted for FAS in 3 ways; i) with only items available for both time points (computers, cars and bedroom occupancy) and ii) as i) but standardised by year; iii) using a summed variable including all items available in each time point standardised by year. As all methods produced consistent results, we present models using version iii. Difficulties in use of FAS items over time are amplified in longer-term analysis, with computer ownership trebling in the period between the 2008 and 2014 surveys. Hence, for analysis of multiple time points, we reran models with adjustment for FAS scores as described above, but additionally with removal of computer ownership items. FAS adjustment made a difference to results only where items for which there had been major social changes (i.e. computer ownership) were not removed or standardised by year. Hence, in all presented results, unadjusted odd ratios are reported without adjustment for FAS.

The proportional odds assumptions for ordinal logistic regressions which assessed changes in exposure to tobacco and e-cigarettes outside public places were violated for e-cigarettes as an outcome, but not smoking. Binary logistic regressions were also therefore conducted for e-cigarettes and smoking. There was a significant increase in exposure to e-cigarettes in public locations [OR = 2.69; CI 95% = 2.29 to 3.17; p<0.001; n = 3,461] and a decrease in smoking exposure in public locations [OR = 0.79; CI 95% = 0.64 to 0.97; p=0.023; n = 3,544].

Table S1: Change over time in perceived smoking norms as well as use, and exposure to, smoking and e-cigarettes for children with no smoking parent figure

|  | **2007**  n (%) | **2008**  n (%) | **2014**  n (%) | **2019**  n (%) |
| --- | --- | --- | --- | --- |
| Pupil’s smoking susceptibility as well as awareness and ever use of tobacco and e-cigarettes | | | | |
| Smoking susceptibility | 76 (9.6) | 96 (11.4) | 63 (7.4) | 112 (8.8) |
| Ever smoked | 13 (1.7) | 6 (0.7) | 7 (0.8) | 4 (0.3) |
| E-cigarette awareness | - | - | 543 (67.0) | 1,063 (82.4) |
| Ever used an e-cigarette | - | - | 28 (3.5) | 36 (2.8) |
| Parental vaping | | | | |
| Parent figure uses e-cigarettes | - | - | 40 (18.6) | 129 (31.2) |
| Exposure tobacco and e-cigarettes in the home | | | | |
| Parent figure who smokes in the home | - | - | - | - |
| Someone was smoking in the home yesterday | 12 (1.5) | 24 (2.9) | 15 (1.8) | 15 (1.2) |
| Smoking rules in home  Full restriction | 593 (84.0) | 650 (87.3) | 683 (91.0) | 1,017 (89.3) |
| Partial restriction | 99 (14.0) | 83 (11.1) | 64 (8.5) | 101 (8.9) |
| No restriction | 14 (2.0) | 12 (1.6) | 4 (0.5) | 21 (1.8) |
| Visitors smoke in the home | 138 (19.9) | 112 (15.2) | 81 (11.0) | 88 (7.2) |
| People use e-cigarettes in the home | - | - | 67 (8.4) | 173 (13.6) |
| Exposure to tobacco and e-cigarettes in a car | | | | |
| Smoking allowed in family car | 26 (3.3) | 28 (3.3) | 8 (1.0) | 18 (1.4) |
| In a car where someone was smoking yesterday | 5 (0.7) | 9 (1.1) | 10 (1.2) | 7 (0.6) |
| Sometimes in a car where people smoke | - | - | 116 (13.7) | 138 (10.7) |
| Someone uses e-cigarettes while I am inside a car | - | - | 43 (5.5) | 106 (8.3) |
| Exposure to smoking and e-cigarettes in public places^b^ | | | | |
| Number of public places children reported seeing smoking in past month | | None | 157 (19.0) | 251 (19.5) |
|  |  | One | 126 (15.2) | 204 (15.8) |
|  |  | Two | 159 (19.2) | 261 (20.2) |
|  |  | Three | 141 (17.0) | 235 (18.2) |
|  |  | Four | 108 (13.0) | 182 (14.1) |
|  |  | Five | 74 (8.9) | 89 (6.9) |
|  |  | Six | 63 (7.6) | 68 (5.3) |
| Number of public places children reported seeing e-cigarettes in past month | | None | 473 (60.6) | 415 (32.4) |
|  |  | One | 69 (8.8) | 166 (13.0) |
|  |  | Two | 55 (7.0) | 211 (16.5) |
|  |  | Three | 55 (7.0) | 194 (15.1) |
|  |  | Four | 54 (6.9) | 130 (10.1) |
|  |  | Five | 34 (4.4) | 75 (5.9) |
|  |  | six | 41 (5.3) | 91 (7.1) |
| Perceived smoking norms | | | | |
| Most adults smoke | 331 (42.0) | 310 (37.0) | 261 (30.7) | 316 (24.8) |
| More than ‘hardly any’ children smoke | 343 (43.6) | 330 (39.3) | 256 (30.2) | 306 (24.3) |
| Mind people smoking around me | 727 (93.4) | 779 (93.3) | 761 (92.7) | 1,083 (84.9) |

Table S2: Change over time in perceived smoking norms as well as use, and exposure to, smoking and e-cigarettes for children with a smoking parent figure

|  | **2007**  n (%) | **2008**  n (%) | **2014**  n (%) | **2019**  n (%) |
| --- | --- | --- | --- | --- |
| Pupil’s smoking susceptibility as well as awareness and ever use of tobacco and e-cigarettes | | | | |
| Smoking susceptibility | 114 (20.5) | 150 (22.5) | 103 (18.0) | 126 (17.7) |
| Ever smoked | 51 (7.3) | 47 (7.1) | 15 (2.7) | 14 (1.9) |
| E-cigarette awareness | - | - | 374 (69.0) | 652 (88.6) |
| Ever used an e-cigarette | - | - | 51 (9.5) | 65 (8.9) |
| Parental vaping | | | | |
| Parent figure uses e-cigarettes | - | - | 175 (81.4) | 285 (68.8) |
| Exposure tobacco and e-cigarettes in the home | | | | |
| Parent figure who smokes in the home | 526 (76.1) | 470 (72.2) | 291 (54.3) | 353 (48.6) |
| Someone was smoking in the home yesterday | 301 (43.3) | 278 (42.4) | 125 (23.0) | 168 (23.0) |
| Smoking rules in home  Full restriction | 188 (30.5) | 178 (30.2) | 241 (48.4) | 269 (40.5) |
| Partial restriction | 256 (41.5) | 265 (45.0) | 209 (42.0) | 330 (49.6) |
| No restriction | 173 (28.0) | 146 (24.8) | 48 (9.6) | 66 (9.9) |
| Visitors smoke in the home | 294 (56.4) | 257 (52.9) | 132 (31.5) | 149 (24.6) |
| People use e-cigarettes in the home | - | - | 132 (24.4) | 230 (32.2) |
| Exposure to tobacco and e-cigarettes in a car | | | | |
| Smoking allowed in family car | 277 (39.5) | 245 (36.8) | 123 (21.6) | 110 (15.0) |
| In a car where someone was smoking yesterday | 94 (13.8) | 91 (13.8) | 44 (7.8) | 34 (4.6) |
| Sometimes in a car where people smoke | - | - | 270 (47.6) | 247 (33.5) |
| Someone uses e-cigarettes while I am inside a car | - | - | 72 (13.4) | 152 (21.4) |
| Exposure to smoking and e-cigarettes in public places | | | | |
| Number of public places children reported seeing smoking in past month | | None | 76 (13.7) | 156 (21.3) |
|  |  | One | 90 (16.3) | 114 (15.6) |
|  |  | Two | 75 (13.6) | 142 (19.4) |
|  |  | Three | 101 (18.3) | 141 (19.3) |
|  |  | Four | 87 (15.7) | 78 (10.7) |
|  |  | Five | 59 (10.7) | 58 (7.9) |
|  |  | Six | 65 (11.8) | 42 (5.8) |
| Number of public places children reported seeing e-cigarettes in past month | | None | 277 (52.0) | 241 (33.2) |
|  |  | One | 53 (10.0) | 108 (14.9) |
|  |  | Two | 48 (9.0) | 102 (14.0) |
|  |  | Three | 45 (8.4) | 84 (11.6) |
|  |  | Four | 39 (7.3) | 72 (9.9) |
|  |  | Five | 32 (6.0) | 53 (7.3) |
|  |  | Six | 39 (7.3) | 67 (9.2) |
| Perceived smoking norms | | | | |
| Most adults smoke | 400 (56.8) | 382 (57.1) | 314 (54.9) | 316 (43.9) |
| More than ‘hardly any’ children smoke | 327 (46.6) | 300 (44.9) | 229 (40.3) | 225 (31.9) |
| Mind people smoking around me | 571 (82.8) | 533 (80.9) | 420 (75.3) | 524 (72.4) |

Table S3: Change in children’s reports of parental dual use of cigarettes and e-cigarettes over time

|  | **2007**  n (%) | **2008**  n (%) | **2014**  n (%) | **2019**  n (%) |
| --- | --- | --- | --- | --- |
| Parent figure dual use  Neither | - | - | 721 (57.2) | 1,144 (57.9) |
| Smokes only | - | - | 324 (25.7) | 419 (21.2) |
| Uses e-cigarettes only | - | - | 40 (3.2) | 129 (6.5) |
| Both | - | - | 175 (13.9) | 285 (14.4) |
